# The Cellular Electrophysiological Properties Underlying Multiplexed Coding in Purkinje Cells

**DOI:** 10.1523/JNEUROSCI.1719-20.2020

**Published:** 2021-03-03

**Authors:** Yunliang Zang, Erik De Schutter

**Affiliations:** ^1^Computational Neuroscience Unit, Okinawa Institute of Science and Technology Graduate University, Okinawa 904-0495, Japan; ^2^Volen Center and Biology Department, Brandeis University, Waltham, Massachusetts 02454

**Keywords:** burst-pause computation, cerebellum, dendritic spikes, linear computation, multiplexed coding, Purkinje cell

## Abstract

Neuronal firing patterns are crucial to underpin circuit level behaviors. In cerebellar Purkinje cells (PCs), both spike rates and pauses are used for behavioral coding, but the cellular mechanisms causing code transitions remain unknown. We use a well-validated PC model to explore the coding strategy that individual PCs use to process parallel fiber (PF) inputs. We find increasing input intensity shifts PCs from linear rate-coders to burst-pause timing-coders by triggering localized dendritic spikes. We validate dendritic spike properties with experimental data, elucidate spiking mechanisms, and predict spiking thresholds with and without inhibition. Both linear and burst-pause computations use individual branches as computational units, which challenges the traditional view of PCs as linear point neurons. Dendritic spike thresholds can be regulated by voltage state, compartmentalized channel modulation, between-branch interaction and synaptic inhibition to expand the dynamic range of linear computation or burst-pause computation. In addition, co-activated PF inputs between branches can modify somatic maximum spike rates and pause durations to make them carry analog signals. Our results provide new insights into the strategies used by individual neurons to expand their capacity of information processing.

**SIGNIFICANCE STATEMENT** Understanding how neurons process information is a fundamental question in neuroscience. Purkinje cells (PCs) were traditionally regarded as linear point neurons. We used computational modeling to unveil their electrophysiological properties underlying the multiplexed coding strategy that is observed during behaviors. We demonstrate that increasing input intensity triggers localized dendritic spikes, shifting PCs from linear rate-coders to burst-pause timing-coders. Both coding strategies work at the level of individual dendritic branches. Our work suggests that PCs have the ability to implement branch-specific multiplexed coding at the cellular level, thereby increasing the capacity of cerebellar coding and learning.

## Introduction

The brain is optimized to process information efficiently using a limited number of neurons in each neural circuit. This is often achieved by multiplexed coding, combining different features of spike trains such as temporal scales, timing, rates and pauses to transmit diverse information, a strategy widely used in sensory and motor systems (Riehle et al., 1997; Panzeri et al., 2010; Gire et al., 2013; Hong et al., 2016; Mease et al., 2017). In the cerebellum, Purkinje cells (PCs) receive sensory and motor information via parallel fiber (PF) synaptic input. PCs form the sole output of the cerebellar cortex and their multiplexed firing patterns are critical in cerebellum-associated behaviors (Herzfeld et al., 2015; Chen et al., 2016; Hong et al., 2016; Brown and Raman, 2018). For example in monkeys, saccadic eye movement velocities are encoded by PC spike rates, but movement onsets are encoded by PC pauses (Hong et al., 2016). However, the cellular mechanisms underlying the transitions between spike rate-coding and pause-coding are unknown.

PCs implementing linear spike rate coding has been well supported by electrophysiological and behavioral studies. In slice recordings, PC spike rates linearly represent PF input strength (Walter and Khodakhah, 2006). Behaviorally, both monkey saccadic eye movement velocities (Herzfeld et al., 2015; Hong et al., 2016) and mouse voluntary whisker movement positions (Chen et al., 2016) can be faithfully replicated by PC average spike rates. Nonetheless, it is confusing how PC dendrites achieve linear coding in the presence of a plethora of nonlinear ion channels, including high threshold P-type Ca^2+^ channels that can generate dendritic spikes (Migliore and Shepherd, 2002; Benton and Raman, 2009; Ohtsuki et al., 2012; Zang et al., 2018).

Dendritic Ca^2+^ spikes can be evoked with clustered PF synaptic input (Llinás et al., 1968; Rancz and Häusser, 2006, 2010). Compared with linear coding, PF dendritic spike properties have been poorly characterized and their functional significance was questioned because their initiation requires strongly clustered PF input. Recent evidence supporting their occurrence *in vivo* (Najafi et al., 2014a,b; Wilms and Häusser, 2015; Roome and Kuhn, 2018) necessitates a deeper study of their initiation mechanisms and properties to understand how they are used by PCs. Because of limited experimental techniques, their initiation thresholds, which constrain their functional feasibility during behaviors, remain unknown. Most of all, although pauses caused by dendritic spikes are shown to “clamp” average somatic outputs (Rancz and Häusser, 2010), what signal they use to transmit timing information and supplement linear rate coding is undetermined. We also need to define their functional units, which determine the computational capacity of a dendritic tree.

Using a well-validated computational model (Zang et al., 2018, 2020), we are able to replicate linear computation and localized PF dendritic spikes in a single neuron for the first time. We validate dendritic spike properties with experimental data, make predictions of their thresholds, and explore their computational units.

## Materials and Methods

All simulations were implemented in NEURON (Hines and Carnevale, 1997). The PC was separated into four parts, axon initial segment, soma, main dendrites and spiny dendrites. The model used here was the same as the original model (Zang et al., 2018, 2020) except some minor changes to current conductances in spiny dendrites. In spiny dendrites, the Kv3 current was decreased by 33%. The Iberiotoxin-sensitive BK current increased by 100% and an iberiotoxin-insensitive BK current (Benton et al., 2013) was added. Although all conclusions reported in this work still hold in our original model, we made these modifications to make PF dendritic spike caused pause durations match experiments (Rancz and Häusser, 2010), without affecting other properties. The simulation code is available from ModelDB (http://modeldb.yale.edu/266864).

**Figure 1.**
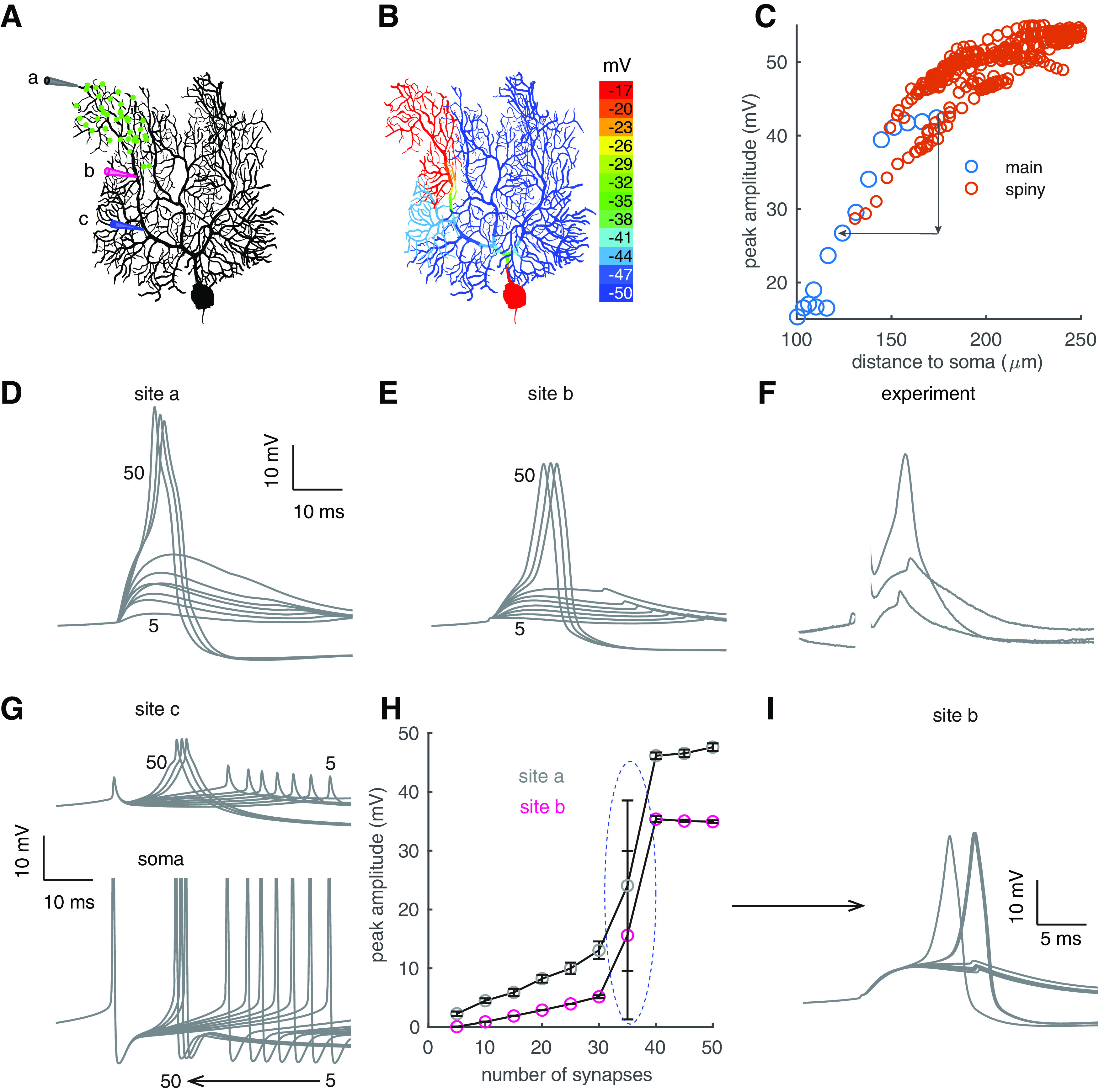
Bimodal dendritic responses in individual PCs. ***A***, Clustered PF synapses within a branch (green dots). Three recording sites: a on the tip, b on the distal main dendrite, and c on the proximal main dendrite. ***B***, Color-coded voltage response peaks to clustered PF input. ***C***, Decayed spike propagation in somapetal direction. Red circles represent sites on spiny dendrites and blue circles represent sites on the main dendrite. Simulated membrane potentials at sites a and b with increasing PF synapses (from 5 to 50) are shown in ***D***, ***E***, respectively. ***F***, Experimentally measured dendritic responses with increasing stimulation intensity compared with site b in ***E*** (data shared by Ede Rancz and Michael Häusser; Rancz and Häusser, 2010). Initial parts of the traces were omitted because of stimulation artifacts. ***G***, Simulated membrane potentials at site c (above) and the soma (bottom, clipped) with increasing PF synapses (from 5 to 50). Note the backpropagated somatic Na^+^ spikes at site c. ***H***, Bimodal dendritic responses at sites a (gray circles) and b (pink circles) versus synchronously activated synapses. Under each condition (the same synaptic number), 10 trials were simulated with randomly distributed synapses. The spike threshold is circled. ***I***, Membrane potentials at site b with 35 PF synapses activated (*n* = 10). Some of the traces are overlapped, resulting in seemingly thicker lines.

**Figure 2.**
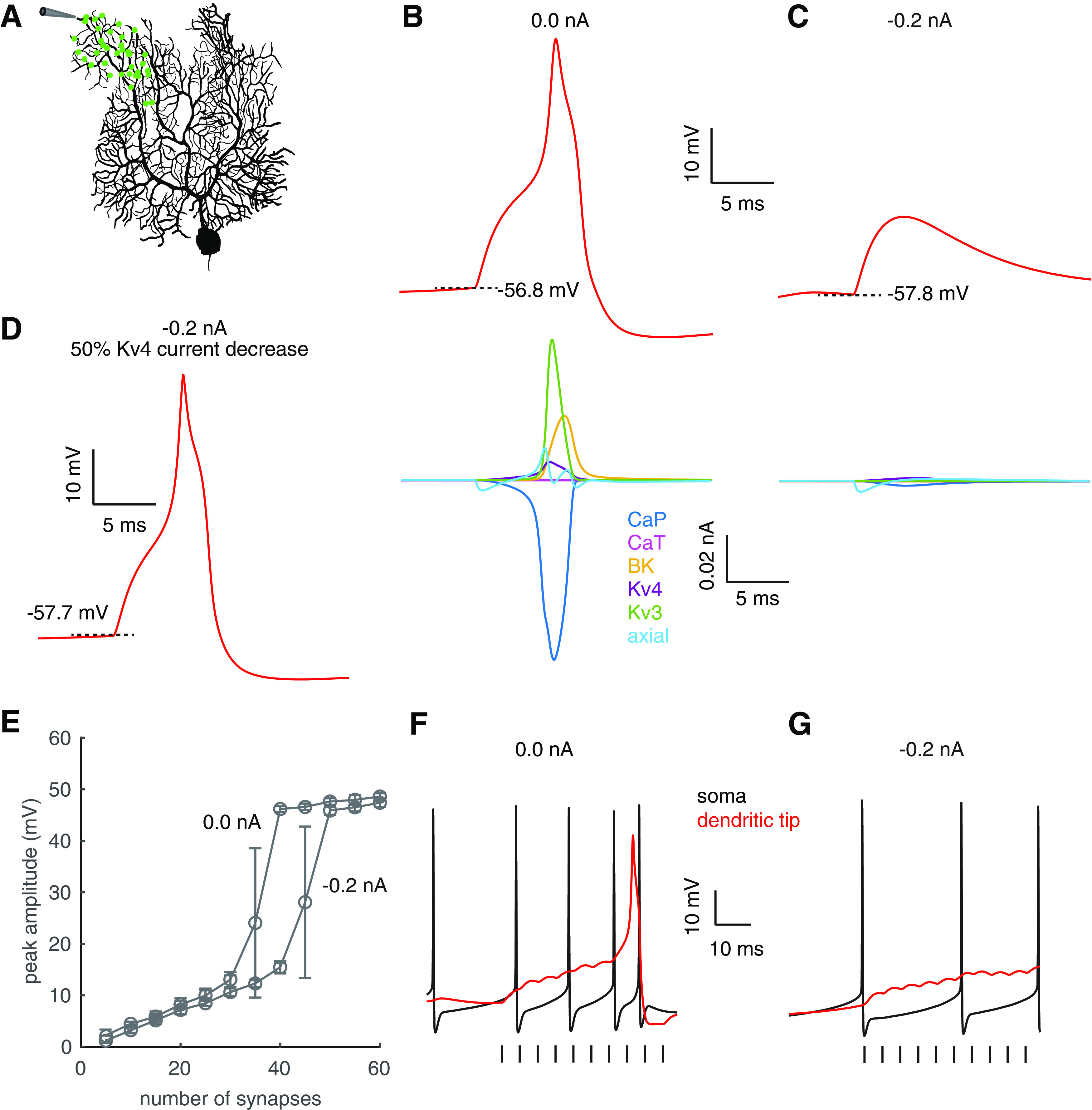
Voltage dependence of dendritic spike threshold. ***A***, A total of 40 PF synapses were distributed in a branch (green dots). Dendritic responses recorded at the tip, where no PF synapses were present, with 0- and −0.2-nA somatic holding currents are shown in ***B***, ***C***, respectively. Top panels show membrane potentials at the dendritic tip, and bottom panels show principal currents under corresponding conditions. The surface area of the segment is 15 μm^2^. ***D***, Recovery of dendritic spiking after locally blocking Kv4 current by 50% (with −0.2-nA somatic holding current). ***E***, Dendritic responses versus number of synapses with 0- and −0.2-nA somatic holding currents. ***F***, ***G***, Somatic (black) and dendritic (red) responses to a train of 10 PF stimuli at 200 Hz. Ten PF synapses were activated during each stimulus. ***F***, 0-nA somatic holding current. ***G***, −0.2-nA somatic holding current. Vertical bars represent the timing of synchronous PF activation.

**Figure 3.**
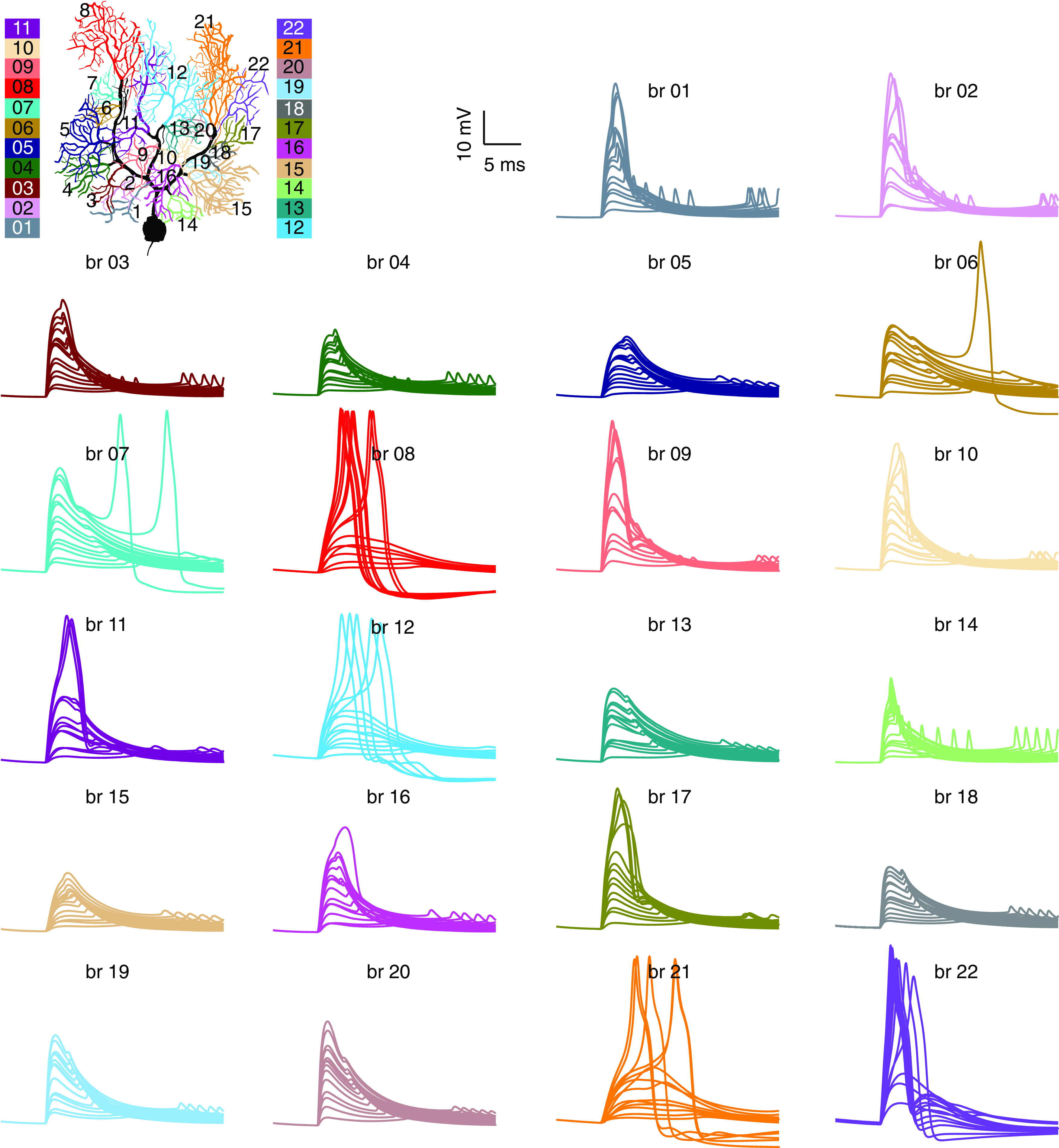
Dendritic responses in individual branches. A total of 22 branches are defined and illustrated by corresponding colors. Black trunk represents the main dendrites. In each panel, traces plot dendritic responses to increasing PF input (from 5 to 70 synapses) in a branch. A repertoire of different response patterns was observed. In branches 8, 12, and 21–22, PF dendric spikes have low thresholds; in branches 1–2, 9–11, and 16–17, spike thresholds are high, making the step increases in input-output curves less obvious; in branches 3–5, 13–15, and 18–20, dendritic spikes cannot be triggered by activating up to 70 PF synapses. In branches 6–7, dendritic spikes occur first in the more excitable distal branch 8 and then trigger spike occurrence in these two branches, as evidenced by delayed spiking.

In Figures 1, 2, simulation results were achieved by distributing synapses within branch 8 (Fig. 3, colored red). We manually grouped PC spiny dendrites into 22 branches along the main dendrites (Fig. 3). For each branch, only one dendritic segment connects it to the parent main dendrite. To simulate clustered PF synaptic input, a defined number of synapses (range 5–70) were randomly distributed on a specified branch and synchronously activated. The synaptic conductance was approximated as a bi-exponential waveform, with 0.3 and 3ms as the rise and decay time constants and peak conductance of 0.5 nS. The reversal potential was 0mV. We mainly recorded membrane potentials at two sites of the model, a dendritic tip (dendritic spike initiation site) and the soma. In Figure 1, to compare with patch-clamp-recorded spike waveforms (Rancz and Häusser, 2006, 2010), a site on the distal main dendrite was recorded, and a site on the proximal main dendrite was recorded to illustrate the decay of PF dendritic spikes. To calculate the axial current in Figure 2*B*,*C*, the segment number was set to three at the tip compartment. We recorded the membrane potentials at 1/2 and 5/6 of the tip compartment. The axial resistance between these two sites was computed by NEURON's built-in function ri(). According to Ohm's law, the axial current was then calculated through dividing the voltage difference between these two sites by the axial resistance (Hines and Carnevale, 1997).

**Figure 4.**
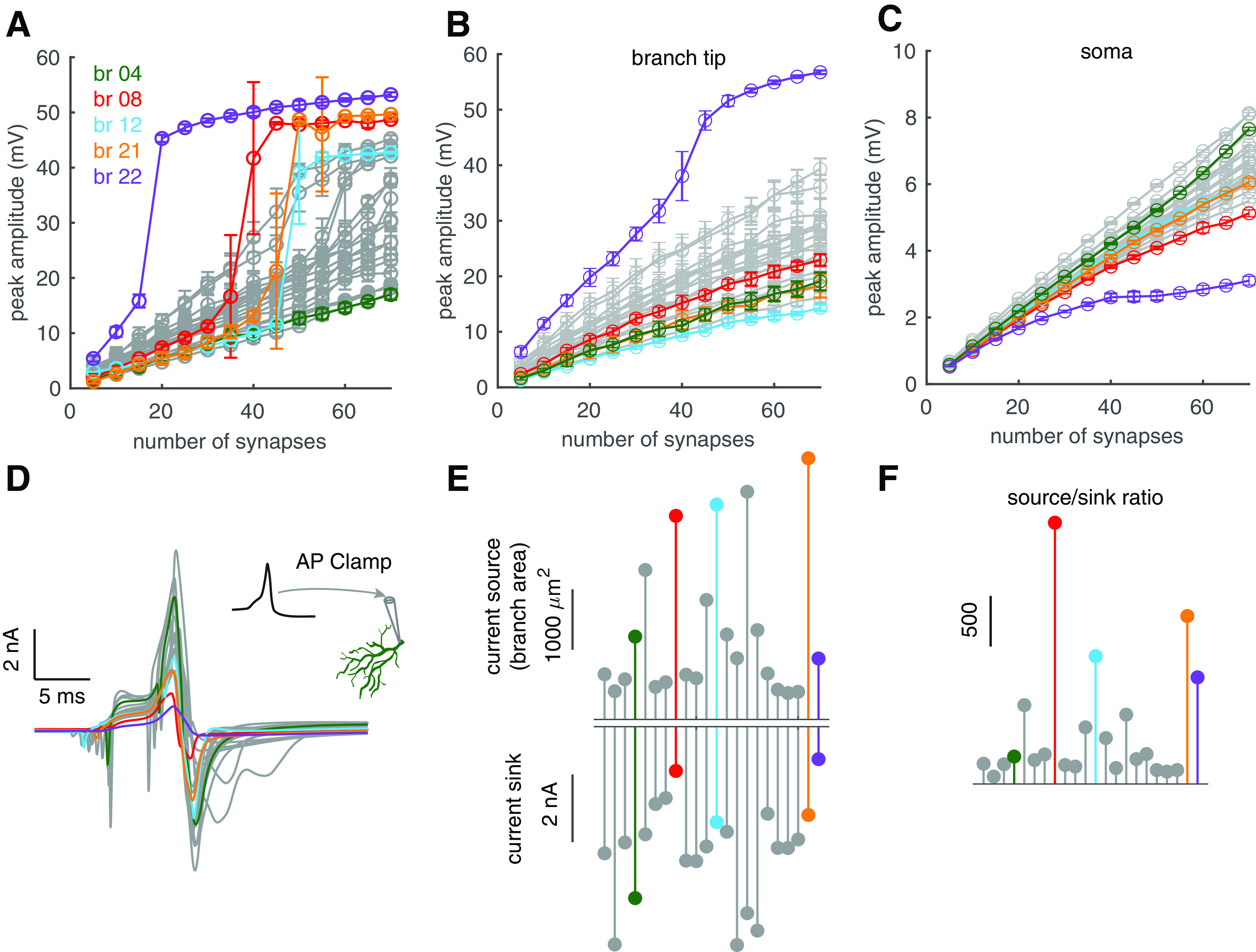
Branch-specific dendritic computation. ***A***, Branch-specific computation and dendritic spike threshold. ***B***, ***C***, Branch-dependent dendritic and somatic responses when the soma was clamped to −71.8 mV. ***D***, Axial currents caused by the sink to other parts when connection segments are clamped with the spike waveform shown in the inset. ***E***, Peak amplitudes of sink-caused axial currents at individual connection segments (bottom) and the surface areas of individual branches (above). Left to right corresponds to branches 1–22. ***F***, Ratios of current sources (branch areas) to current sinks. Except branches 4, 8, 12, 21, and 22, results in other branches were plotted as gray traces in all panels. In ***A***, ***D–F***, there is no holding current in the model.

**Figure 5.**
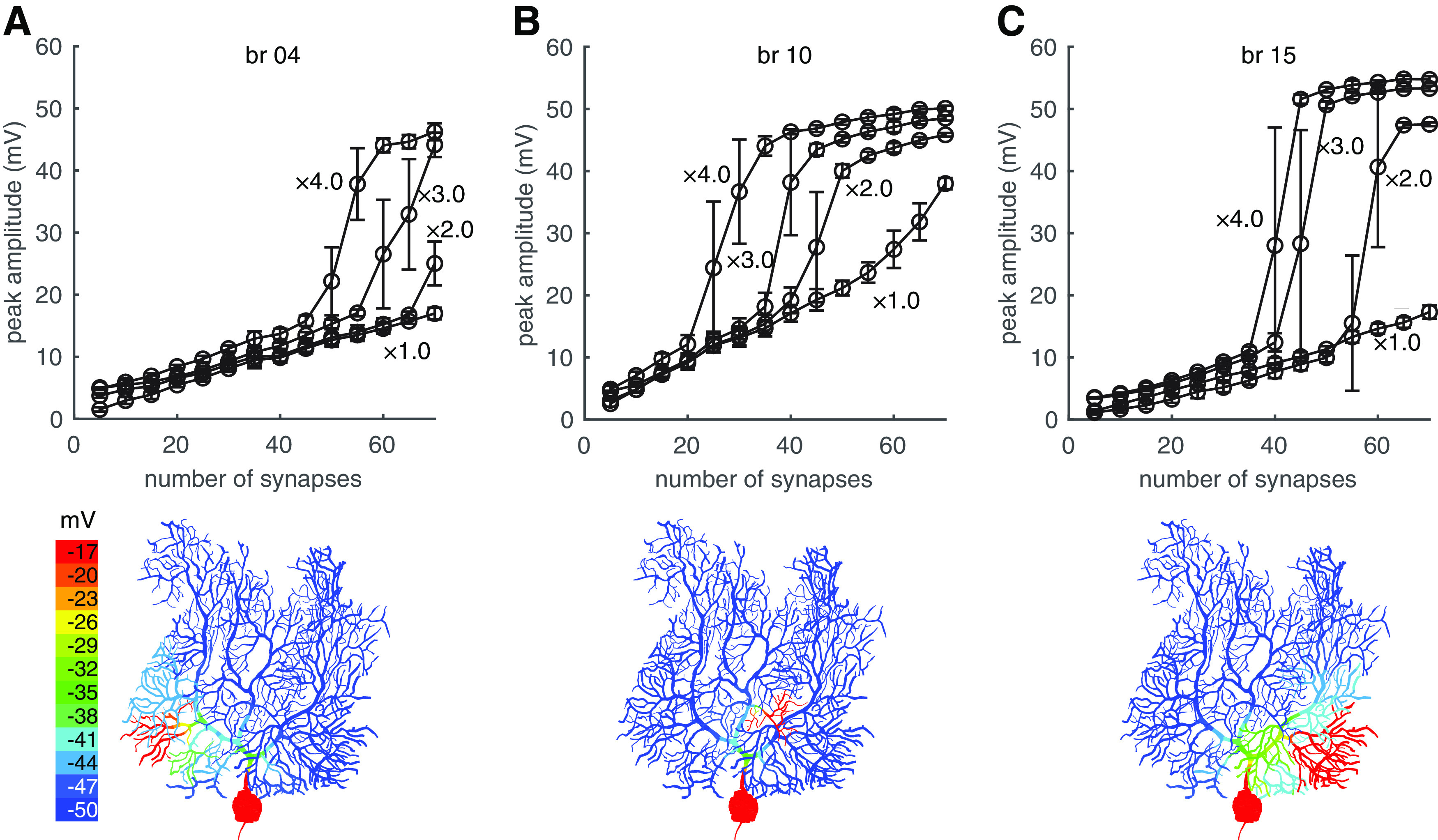
Dendritic branch excitability with segregated clustered PF inputs. The same number of PF synapses in branch 8 and another branch *i* were simultaneously activated. The PC dendritic tree (Fig. 3) is composed of three main limbs from left to right. Branches 1–8 within the left main limb; branches 9–13 within the middle main limb; branches 14–22 within the right main limb. ***A–C*,** Dendritic responses in branch 8. They show how dendritic responses in branch *i* (1–7, 9–22) regulate the excitability of branch 8. For example, when the number of synapses is 40, “8” means there are 40 synapses only in branch 8; “8&*i*” means 40 PF synapses distributed in branch 8 and 40 synapses in branch *i*. ***A***, Co-activated PF inputs are within the same main limb. ***B***, ***C***, Co-activated PF inputs are in different main limbs. ***D–F***, Dendritic responses in branch *i* (4–7, 11–13, 16, 17, 21, 22). They show how dendritic responses in branch 8 regulate the excitability of branch *i*. “8&*i*” has the same meaning as in ***A–C***; “*i*” means there are only clustered inputs in branch *i*. For ***D–F***, only branches exhibiting nonlinear responses are shown.

**Figure 6.**
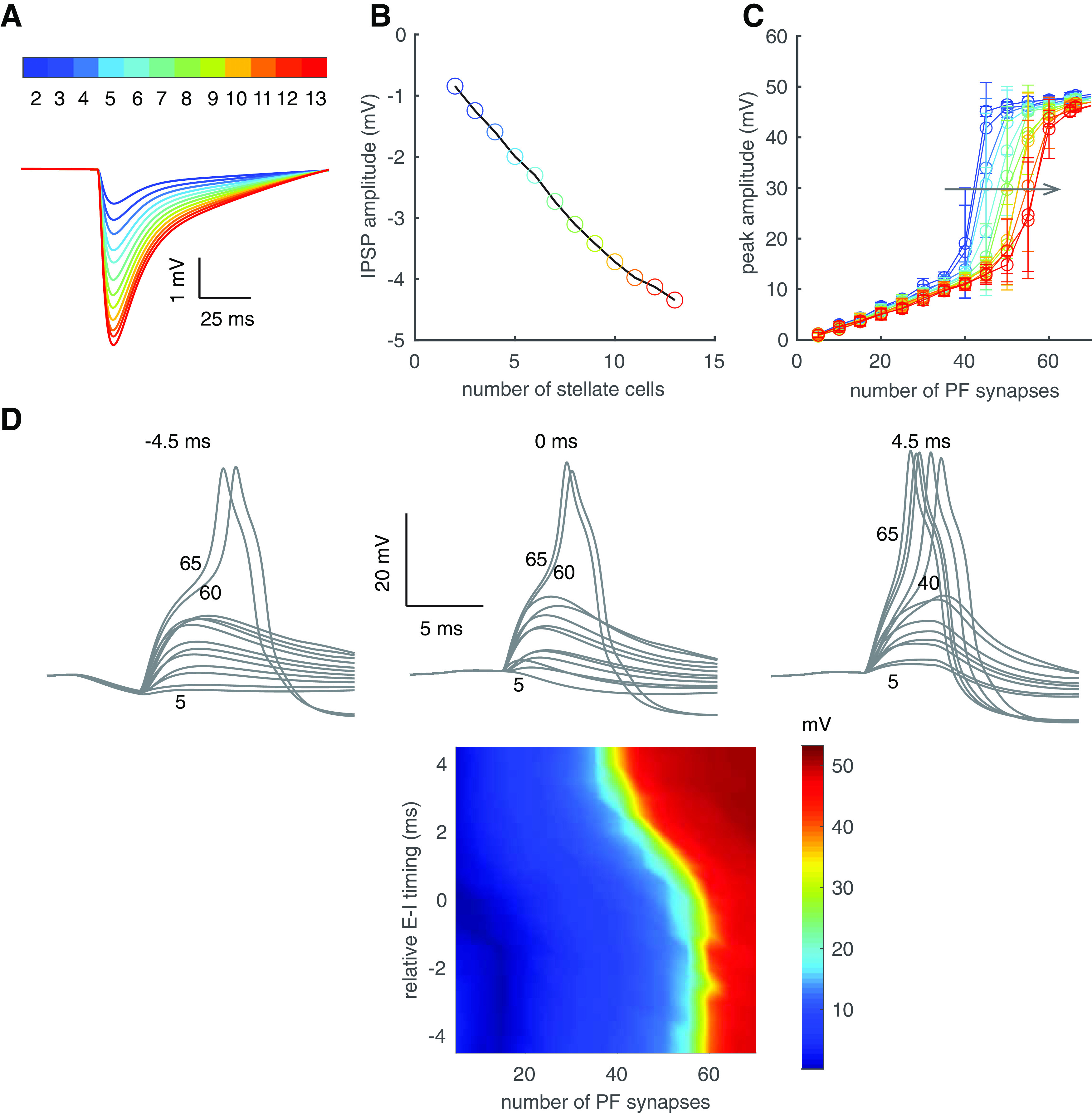
Enhanced branch excitability by locally increased channel densities. ***A–C***, Dendritic responses in branches 4, 10, and 15, respectively. The channel densities in each branch are scaled up to two, three, and four times of original values for different traces. Above panels show dendritic responses versus number of synapses. Bottom panels show the localization of dendric responses when channel densities in corresponding branches were increased to four times of original values.

In all simulations under *in vitro* condition, the PC model fired spontaneously without a holding current, except in following figures. To explore the voltage dependence of the dendritic spike threshold, −0.2 nA was injected into the soma in Figure 2. To illustrate branch-specific PF responses at dendrites and the soma in Figure 4*B*,*C*, the somatic membrane potential was clamped to −71.8 mV by a −0.4-nA holding current. To mimic PF bursting during sensory stimuli (Chadderton et al., 2004), a train of 10 stimuli at 200 Hz (5-ms intervals) were applied to activate PF synapses in Figure 2*F*,*G*. For each stimulus, 10 PF synapses randomly distributed in branch 8 were synchronously activated. To uncover the intrinsic heterogeneity of dendritic spike thresholds in each branch, we used a dendritic spike recorded at the distal main dendrite of branch 8 to clamp membrane potentials of each connection segment (connecting a branch to its parent dendrite) separately in Figure 4*D–F*. The axial currents in Figure 4*D* were computed in the same way as in Figure 2*B*,*C*. Rather than using membrane potential peaks, a “step” increase in input-output curves was used to define the regenerative dendritic spike occurrence. We used the ratio of dendritic branch area (“source,” measuring available Ca^2+^ channels in the branch activated by PFs) to corresponding axial current peak (current “sink” to the other part of the dendritic tree) as the measure to quantify branch excitability. To simplify segregated clustered PF synaptic inputs onto PCs in Figures 5, the same number of PF synapses were simultaneously activated in two branches that we explored. Interactions between more branches were not explored. To explore how heterogeneous distribution of ion channels affects the excitability of individual branches, we scaled up their channel densities from one to four times to increase the source. When channel densities were scaled up to four times, nearly all of the branches can generate low threshold dendritic spikes, but we only showed branches 4, 10, and 15 as examples in Figure 6.

**Figure 7.**
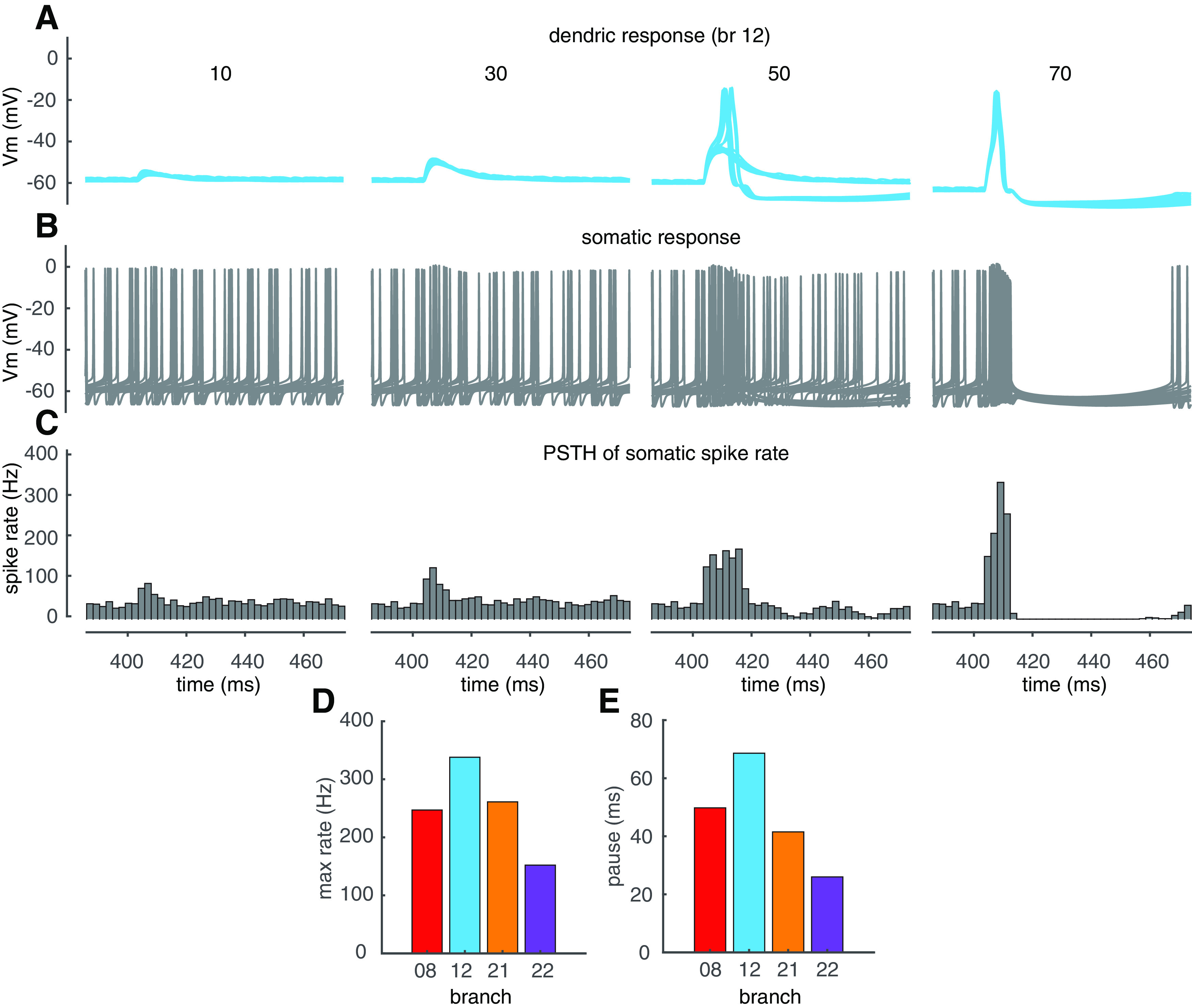
Effect of inhibition on dendritic spike thresholds. ***A***, Dendritic tip IPSPs with increased inhibition. ***B***, IPSP amplitudes linearly increase with inhibition from more stellate cells. In ***A***, ***B***, the model was silenced by somatic holding current, with the soma to −71.8 mV and the dendrite to −67.5 mV. **C**. Inhibition right shifts dendritic spike thresholds (FFI delay is 1.4 ms). For ***A–C***, color codes the number of activated stellate cells (each stellate cell form 16 synapses onto the PC), defined by the color bar in panel ***A***. ***D***, Preceding inhibition preferentially inhibits spike initiation. Example dendritic responses when inhibition occurs −4.5, 0, and 4.5 ms relative to PF excitation are shown on top from left to right. Numbers represent activated PF synapses with inhibition from eight activated stellate cells. Bottom plot summarizes the relationship between dendritic response amplitudes, inhibition timing and number of activated PF synapses (inhibition from eight stellate cells).

A typical feed-forward inhibition (FFI) delay of 1.4 ms (Mittmann et al., 2005) was used in Figure 7*C* to explore how inhibition regulates the dendritic spike threshold. Each stellate cell forms 16 synapses onto the spiny dendrites of the PC model. Inhibitory synaptic conductance was approximated as a bi-exponential waveform, with 1 and 8 ms as the rise and decay time constants. The peak conductance of synaptic input from 1 stellate cell to the PC is 1 nS. The reversal potential is −85 mV (Mittmann and Häusser, 2007). To explore how temporal inhibition regulates the dendritic spike threshold, we systematically varied the timing of inhibition from −4.5 ms (before) to 4.5 ms (after) relative to PF activation.

**Figure 8.**
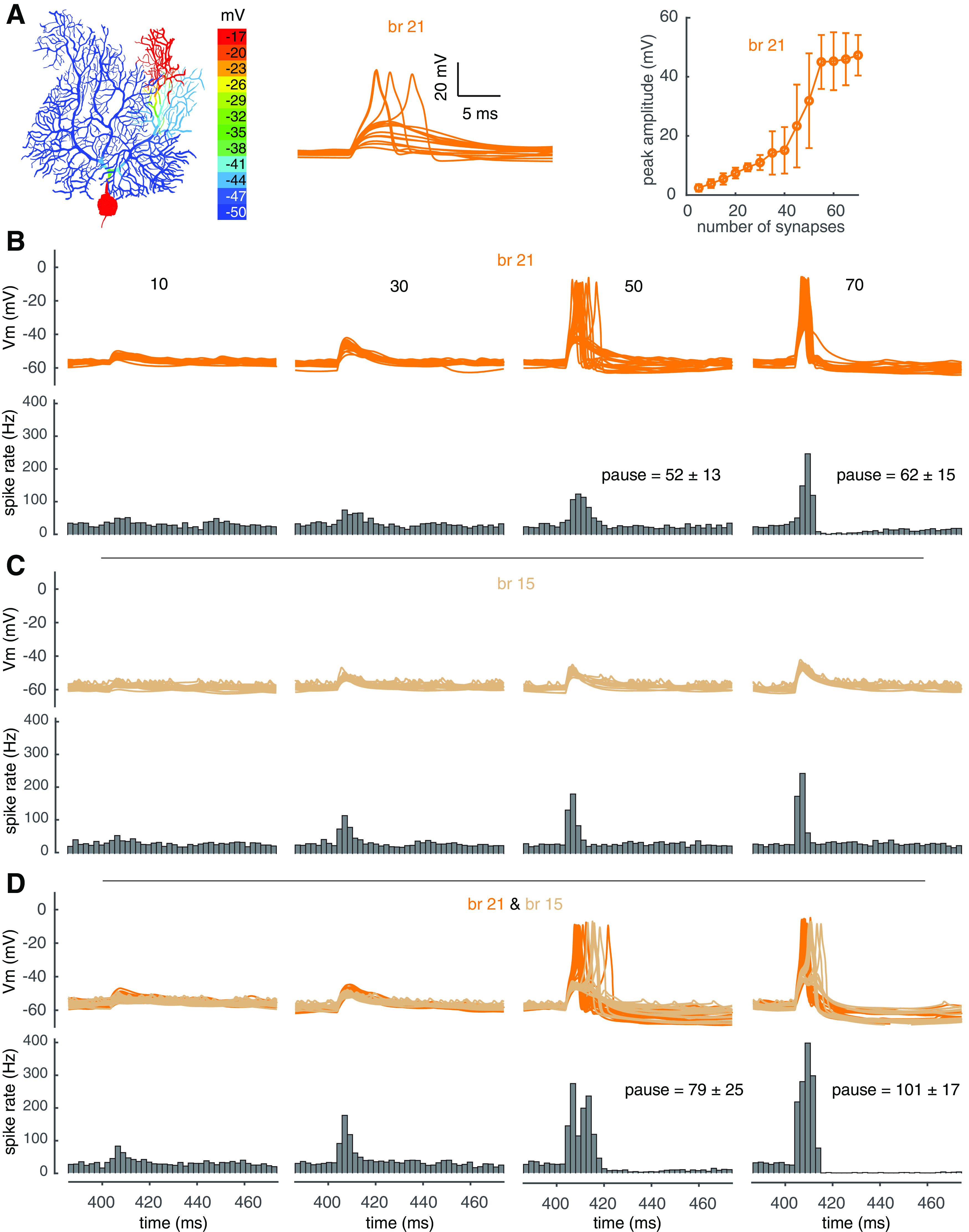
Dendritic spikes trigger branch-specific somatic burst-pause sequences. ***A***, ***B***, Membrane potentials at the dendritic tip and the soma, respectively (20 trials randomly selected from 500 simulation trials). ***C***, PSTHs of somatic spiking rate (bin size 2 ms, 500 simulation trials with randomly distributed PF synapses and disturbed somatic spike timing). From left to right in ***A–C***, PF synapses increase from 10 to 70, and simulation results were obtained in branch 12 (Fig. 3). ***D***, Branch-specific somatic maximum spike rates caused by localized dendritic spikes. ***E***, Branch-specific somatic pause durations following bursts.

**Figure 9.**
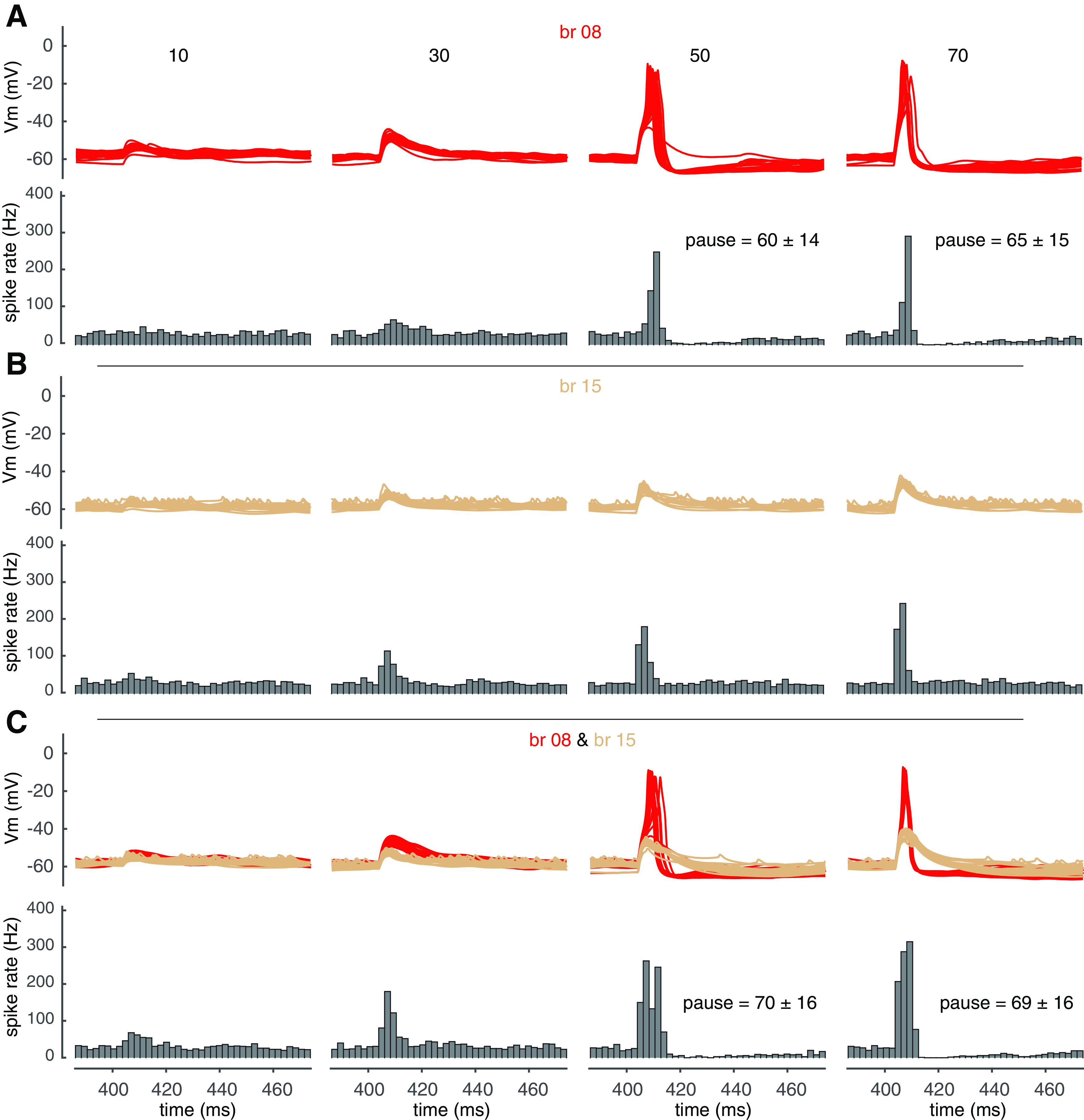
The effect of PF dendritic spikes on somatic output under *in vivo* condition. ***A***, Localized PF dendric spike in branch 21. Left shows the color-coded dendritic response with 65 PF synapses activated; middle shows dendritic membrane potentials with increasing PF synapses (5–70); right shows dendric responses versus activated PF synapses. ***B***, Dendritic responses (above) and PSTHs of somatic spiking rate (bottom) with increasing PF input in branch 21. ***C***, Dendritic responses (above) and PSTHs of somatic spiking rate (bottom) with increasing PF input in branch 15. ***D***, Dendritic responses (above, orange for branch 21 and wheat for branch 15) and PSTHs of somatic spiking rate (bottom) with the same number of PF synapses in each branch. For dendritic responses in ***B–D***, 20 trials were shown for each condition.

**Figure 10.**
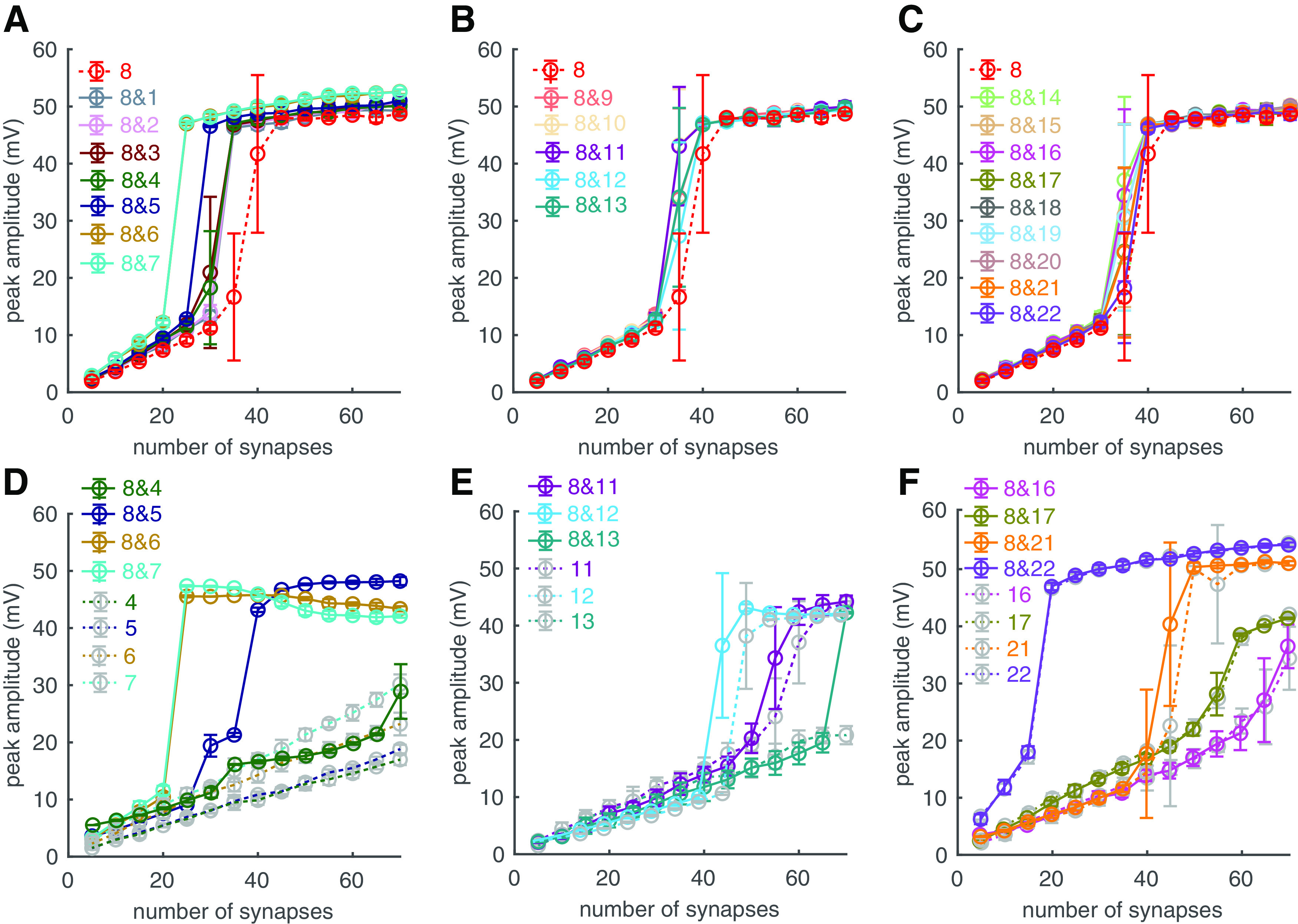
Modulation of somatic output by co-activated PF inputs under *in vivo* condition. Here, branch 15 still linearly integrates PF input with co-activated branch 8. ***A***, Dendritic responses (above) and PSTHs of somatic spiking rate (bottom) with increasing PF input in branch 8. ***B***, Dendritic responses (above) and PSTHs of somatic spiking rate (bottom) with increasing PF input in branch 15. ***C***, Dendritic responses (above, red for branch 8 and wheat for branch 15) and PSTHs of somatic spiking rate (bottom) with the same number of PF inputs in each branch. For dendritic responses under each condition in ***A–C***, 20 trials from 500 simulations were shown.

To obtain peristimulus time histograms (PSTHs) of somatic spiking, 500 trials were simulated for each condition in Figures 8–10. During each trial, synapses were randomly distributed and somatic spike timing was randomly disturbed by a holding current of different length at the beginning of the simulation. To mimic background synaptic inputs in PCs, 160 basket cell synapses (from four basket cells) were evenly distributed on the soma and axon initial segment. A total of 144 stellate cell synapses (from nine stellate cells) were evenly distributed on spiny dendrites. They both generate spontaneous synaptic inputs with mean spike rates of 14.4 Hz sampled from a Poisson distribution. 2000 PF synapses were evenly distributed on spiny dendrites with a mean spike rate of 0.135 Hz sampled from a Poisson distribution. With these background inputs, dendritic membrane potential variations are close to *in vivo* recordings (Jelitai et al., 2016). We did not include spontaneous climbing fiber (CF) input.

## Results

### Localized dendritic spike initiation

To mimic clustered PF input (Rancz and Häusser, 2006, 2010), 40 synapses were randomly distributed within a dendritic branch of the PC model firing spontaneously at 40 Hz (Fig. 1*A*). Once these synapses are simultaneously activated, dendritic spikes occur and are spatially constrained (Fig. 1*B*). Dendritic spikes are initiated at distal tips and propagate to proximal parts with reducing amplitudes (Fig. 1*C*). Close to the 69 ± 3% decrease over 54 ± 11-μm distance observed experimentally (Rancz and Häusser, 2006), the peaks of dendritic spikes in the model decrease by 55% over 50-μm distance when propagating to the soma (Fig. 1*C*). The passive propagation is because of the large impedance mismatch in PC dendrites (Vetter et al., 2001). Next, we analyzed spike properties and tested their thresholds, i.e., the number of synapses required for dendritic spike initiation. With weak input, dendritic response amplitudes linearly increase with the number of activated PF synapses (Fig. 1*D*,*E*,*H*), suggesting a linear computation in PC dendrites. Once a threshold of 35 synapses is reached, dendritic response amplitudes show a step increase and maintain a fixed value even with stronger input (Rancz and Häusser, 2006; Fig. 1*H*). Dendritic spikes show a plateau during initial rising phase and then an accelerating depolarization, in agreement with patch-clamp-recorded spike shapes (compare Fig. 1*E* and *F*, both recorded on distal main dendrites). Because of severe decay, PF triggered dendritic responses are nearly indiscernible from back-propagated somatic spikes at proximal dendrites, but dendritic responses significantly advance the timing of the following somatic spike (Fig. 1*G*). With 35 synapses activated, random variation of synaptic positions makes dendritic responses “all-or-none” (Fig. 1*I*), explaining the large variance of dendritic response amplitudes at the threshold (Fig. 1*H*). Conversely, activating 40 synapses reliably triggers dendritic spikes.

### Voltage dependence of dendritic spike thresholds

The number of PF synapses required to trigger dendritic spikes determines their functional availability during behaviors. Can PF dendritic spike thresholds be dynamically regulated by voltage states like complex spikes are (Zang et al., 2018)? We first analyzed the principal currents causing a PF-evoked dendritic spike at the dendritic site shown in Figure 2*A*. After a stimulation, synaptic input depolarizes neighboring dendritic segments and provides positive axial current into this site (Fig. 2*B*), which then depolarizes the site to reach the activation threshold of P-type Ca^2+^ current. Once the Ca^2+^ current takes over the depolarization, the polarity of the axial current becomes negative. The spike is repolarized mainly by Kv3 and large conductance Ca^2+^-activated K^+^ current (BK). Note that Ca^2+^ channels can also be activated by PF synaptic current directly (data not shown). The dendritic membrane potential was −56.8 mV when PF synapses were activated in the spontaneously firing model. Once it was hyperpolarized to −57.8 mV by −0.2-nA somatic holding current, 40 PF synapses no longer trigger dendritic spikes (Fig. 2*C*). The failure of dendritic spiking by hyperpolarization agrees with previous experiments (Rancz and Häusser, 2006). Here, we analyzed the ionic mechanism. PF-evoked depolarization in other parts of this branch still provides positive axial current into this site, but fails to reach the activation threshold of Ca^2+^ current and ionic channels are barely activated (Fig. 2*C*). Two factors cause the failure. The first is simply the hyperpolarized basal membrane potential, which requires a larger depolarization to reach the activation threshold of Ca^2+^ current. The second is a larger availability of Kv4 current at the hyperpolarized membrane potential, which also elevates the spike threshold. The role of Kv4 current in regulating spike threshold is supported by the dendritic spike recovery after locally blocking it by 50% (Fig. 2*D*). Compared with spontaneously firing condition, −0.2-nA holding current increases the spike threshold from 35 to 45 PF synapses. Accordingly, the dynamic range of linear computation is expanded (Fig. 2*E*).

*In vivo*, granule cells respond to sensory stimuli with bursts of spikes (Chadderton et al., 2004), which implies fewer simultaneously activated PF synapses may be required to trigger dendritic spikes. In the same branch as previously stimulated, we exerted a train of 10 PF stimuli at 200 Hz, with just 10 PF synapses activated during each stimulus. In the spontaneously firing model, a dendritic spike occurs in the stimulated branch (Fig. 2*F*), but hyperpolarization inhibits its occurrence (the model fires at 12 Hz with −0.2-nA somatic holding current; Fig. 2*G*).

### Branch-specific dendritic computation

The number of computational units determines the coding capacity of dendritic trees. We defined 22 branches contacting the main dendrites and evaluated their responses to clustered PF inputs (Fig. 3). With weak inputs, dendritic responses linearly increase with PF synapses in all branches. With stronger PF inputs (examined up to 70 PF synapses), some branches such as branch 12, show obvious bimodal “linear-step-plateau” responses, while others such as branch 4 do not. Responses in branch 4 always linearly increase with the number of activated synapses without either step increases in gain curves or regenerative dendritic spike occurrence (Figs. 3, 4*A*). The input-output curves of all branches are shown in Figure 4*A*, showing that dendritic responses are branch dependent both in the range of linear integration and dendritic spike initiation. We next examined how dendritic linear integration regulates somatic output. The soma was clamped to −71.8 mV to record somatic responses when dendrites implement linear computations in Figure 4*B*,*C*. We find that somatic depolarizations still differ when branch-dependent dendritic responses propagate to the soma after drastic dendritic filtering. The effect of dendritic spikes on somatic output will be explored in later sections.

In our model, channel densities in spiny dendrites are homogeneous (Zang et al., 2018). Here, we investigated why some branches have higher excitability and show obvious bimodal responses, but others do not. As shown in Figure 2*B*, the current source, Ca^2+^ current within a branch needs to overcome the current sink to other parts to reach the spike threshold. However, if a branch is either too small (equivalently little source) or strongly coupled to other parts of the dendrite (small axial resistance causing a large current sink), Ca^2+^ current within the branch is incapable of overcoming the current sink to generate dendritic spikes. To demonstrate this theory, we assumed that dendritic spikes occurred in every branch. We used a previously recorded dendritic spike waveform to separately clamp the segments connecting individual branches and their parent main dendrites (Fig. 4*D*). The spike waveform and an example of connection segment in branch 4 are shown in the inset. We recorded axial currents at connection segments to measure the current sink if dendritic spikes would occur there. Axial current peak amplitudes in individual branches are shown in Figure 4*E*, bottom. Although branches showing obvious bimodal responses always have small values of current sink, they are not the sole determining factor as manifested by similarly small values in some other branches. The same argument applies to the size of the current source, despite large surface areas for excitable branches (Fig. 4*E*, above). Figure 4*F* clearly demonstrates that it is the “source/sink” ratio that determines the excitability of a branch. Branches 8, 12, 21, and 22 have low spike thresholds and exhibit the most obvious linear-step-plateau responses (Fig. 4*A*), because of their largest source/sink ratios. In other branches, smaller source/sink ratios cause either higher spike thresholds making step increases less obvious in input-output curves or the failure of regenerative dendritic spikes (Figs. 3, 4*A*). The step increase in the input-output curve is still absent in less excitable branches such as branch 4 even when increasing the range of PF synapse activation to 100 (data not shown).

### Interactions between branches

In previous simulations, individual branches show different responsiveness to PF input and only some branches show obvious linear-step-plateau responses. The simulation protocol we used is consistent with previous *in vitro* experiments (Rancz and Häusser, 2006, 2010). However, with sensory stimuli, PFs can convey segregated clustered synaptic inputs onto different dendritic branches (Wilms and Häusser, 2015). It is possible that co-activation of PF synapses in different branches can affect the excitability of each branch. In our model, dendritic branches are distributed along main dendrites in three limbs (Fig. 3, black trunk). We took branch 8, which is within the left main limb and shows obvious linear-step-plateau responses, as an example. We find that co-activated PF inputs within the same main limb can efficiently lower the dendritic spike threshold of branch 8 (Fig. 5*A*), while co-activated PF inputs in other main limbs only slightly increase its excitability (Fig. 5*B*,*C*). Similarly, dendritic responses in branch 8 are more efficient at facilitating the excitability of dendrites within the same main limb (Fig. 5*D–F*). With co-activated PF input in branch 8, branches 5–7 show obvious linear-step-plateau responses at low thresholds and branch 4 at a high threshold, but all these nonlinear responses are absent when they receive only single clustered inputs (Fig. 5*D*). In contrast, co-activated input in branch 8 is less efficient at facilitating the excitability of branches in other limbs. In the middle limb, the spiking thresholds of branches 11 and 12 are slightly decreased and branch 13 only show obvious nonlinear responses with >65 synapses (Fig. 5*E*). The effect of facilitation is even smaller on branches in the right limb (Fig. 5*F*). Other branches do not show obvious bimodal dendritic responses with co-activated input in branch 8, and therefore their results are not shown.

### Effect of heterogeneous channel density on dendritic spike threshold

There is a possibility that channel densities in different branches are heterogenous (Ohtsuki et al., 2012). We systematically tested whether dendritic spikes can occur in each branch at low thresholds, if local channel densities are scaled up to increase the source (Fig. 6). When local channel densities are increased to four times of original values, nearly all of the branches show obvious linear-step-plateau responses at low thresholds except branches 18–20. Example dendritic responses in branches 4, 10, and 15 are shown in Figure 6. These spikes are still localized within their branches. Globally increasing dendritic channel densities in our model causes spontaneous Na^+^-Ca^2+^ bursting and dendritic spike propagation (data not shown), which are against experimental observations (Rancz and Häusser, 2006, 2010).

### Effect of inhibition on dendritic spike threshold

When PCs receive excitatory input from PFs, they also receive inhibition activated by PFs because of a typical FFI circuit (Mittmann et al., 2005; Gaffield et al., 2018; Rowan et al., 2018; Arlt and Häusser, 2020). According to electrophysiological data (Häusser and Clark, 1997; Mittmann and Häusser, 2007; Wilms and Häusser, 2015), the number of inhibitory neurons targeting the same PC is ∼10 at most. Stellate cells target mainly spiny dendrites, and therefore we studied such inputs (2–13 in simulations) to test the effect of inhibition on dendritic spike thresholds. IPSPs recorded at the dendritic tip linearly increase with inhibition strength at resting condition (Fig. 7*A*,*B*). In the spontaneously firing model, inhibition was recruited 1.4 ms after PF inputs, a typical EPSC-IPSC delay when a PC and its presynaptic inhibitory neurons are activated by the same PFs (Mittmann et al., 2005). Both PF synapses and inhibitory synapses were randomly distributed in branch 8. As evidenced by the right shifted input-output curve, the linear computation range is expanded and the dendritic spike threshold increases from 35 to 60 PF synapses when inhibition gets stronger (Fig. 7*C*).

Inhibition onto a PC may be recruited by PFs that do not contact this PC. Thus, the timing of inhibition relative to excitation is a critical factor to regulate dendritic integration. Here, we simulated PF-evoked dendritic responses with inhibition (8 stellate cells) sliding from 4.5 ms before to 4.5 ms after excitatory inputs (Fig. 7*D*). Preceding inhibition depresses dendritic responses more efficiently compared with posterior inhibition. With the inhibition from 8 stellate cells at 4.5 ms before PF inputs, the spike threshold increases from 35 (no inhibition) to 60 PF synapses. The dendritic spike threshold keeps relatively unchanged when preceding inhibition approaches the timing of PF inputs. Once inhibition occurs after PF inputs, the effect of inhibition gets weaker with the time delay. When inhibition is activated 1.5 ms after PF inputs (typical FFI delay being 1.4 ms; Mittmann et al., 2005), the threshold only increases from 35 to 50 PF synapses. The more efficient preceding inhibition can be explained by a “shunting” effect: an overlap of IPSC and EPSC reduces the local impedance (Stuart et al., 2016). When the FFI delay increases to 4.5 ms, inhibition no longer affects the spike threshold, since dendritic spikes occur before inhibition.

### The effect of PF dendritic spikes on somatic output

How do PF-evoked dendritic spikes regulate somatic output? Example dendritic and somatic responses are shown in Figure 8*A*,*B*. The PSTHs of somatic spiking rate (Chen et al., 2016) is shown in Figure 8*C*. Below the dendritic spike threshold (50 synapses in branch 12), dendritic responses increase with PF input and elevate somatic spiking rate by triggering a burst clustered to PF input timing. Once dendritic spikes occur, the somatic burst is followed by a pause. When dendritic spikes are reliably triggered, the maximum spike rates during bursts and the durations of pauses are relatively invariant because of the all-or-none nature of dendritic spikes. These results suggest that PF dendritic spikes can trigger reliable burst-pause sequences. Do dendritic spikes evoked in different branches carry identical burst-pause information at the soma? We summarized dendritic spike-caused somatic maximum spike rates during bursts and the durations of pauses in 4 branches that show the most obvious linear-step-plateau responses (branches 8, 12, 21, and 22; Fig. 8*D*,*E*). Simulation results show individual branches carry different pairs of maximum spiking rates and pause durations. Dendritic spiking in branch 12 regulates somatic output more efficiently by triggering the maximum spike rate to 338 Hz and the pause to 69 ms. In contrast, branch 22 is less efficient at regulating somatic output. Although spike amplitudes are similar in different branches, they have different electronic distances to the soma and therefore undergo different degrees of filtering before they reach the soma and regulate somatic output.

### Burst-pause coding under *in vivo* condition

*In vivo*, PCs receive synaptic inputs even when animals are resting (Chen et al., 2017; Roome and Kuhn, 2018). Here, we tested the properties of PF dendritic spikes with “background” synaptic inputs, relating it to experimental observations (Najafi et al., 2014a,b; Wilms and Häusser, 2015; Roome and Kuhn, 2018). Background excitatory input was mimicked by distributing PF synapses evenly onto spiny dendrites, and inhibitory inputs were mimicked by distributing basket cell synapses onto the soma and axon initial segment, and stellate cell synapses onto spiny dendrites (details in Materials and Methods). Figure 9*A* displays an example of localized spiking in branch 21, showing obvious linear-step-plateau responses to increased PF input. With 50 PF synapses, dendritic spikes start to occur in some trials, causing increased somatic maximum spike rate and the appearance of pausing in somatic spikes. When the input increases to 70 synapses, dendritic spikes occur more robustly and therefore both the somatic maximum spike rate and the pause duration increase further (Fig. 9*B*). The “noisier” dendritic membrane potentials are because of background synaptic inputs. The “noisier” dendritic membrane potentials are because of background synaptic inputs. In contrast, in branch 15, both dendritic responses and somatic maximum spike rates increase with PF input and lack pauses following the bursts within the tested range of PF input (Fig. 9*C*).

With segregated clustered inputs between branches, original linear-coding branches can transition into burst-pause-coding branches, and the interaction between branch 21 and branch 15 falls into this category. With co-activated inputs in these two branches, the spike threshold of branch 21 is significantly lowered (within the same limb) and 50 PF synapses reliably trigger dendritic spikes in it. Meanwhile, dendritic spikes also occur in branch 15 in some trials. Therefore, both the somatic maximum spike rate and the pause duration increase significantly compared with the values caused by dendritic spiking only in branch 21 (compare Fig. 9*B* and *D*). With stronger inputs, the somatic maximum spike rate and pause duration increase further because of more reliable dendritic spiking in branch 15. We also simulated co-activated PF inputs in branches 8 and 15 as another example in Figure 10. As shown in Figure 10*A*, branch 8 is a burst-pause-coding branch, and branch 15 remains a linear coder with co-activated input in branch 8 (Fig. 10*B*,*C*). Linear integration in branch 15 makes the somatic maximum spike rate increase further with input strength, but keeps the pause duration relatively unchanged when compared with the values caused by dendritic spiking only in branch 8. This is because somatic burst is clustered to the PF input, which shows a large temporal segregation with the following pause caused by dendritic spiking (compare Fig. 10*A* and *B*). Our results show that co-activated PF inputs between branches can finely modulate the somatic burst-pause information caused by dendritic spikes.

## Discussion

**Figure 11.**
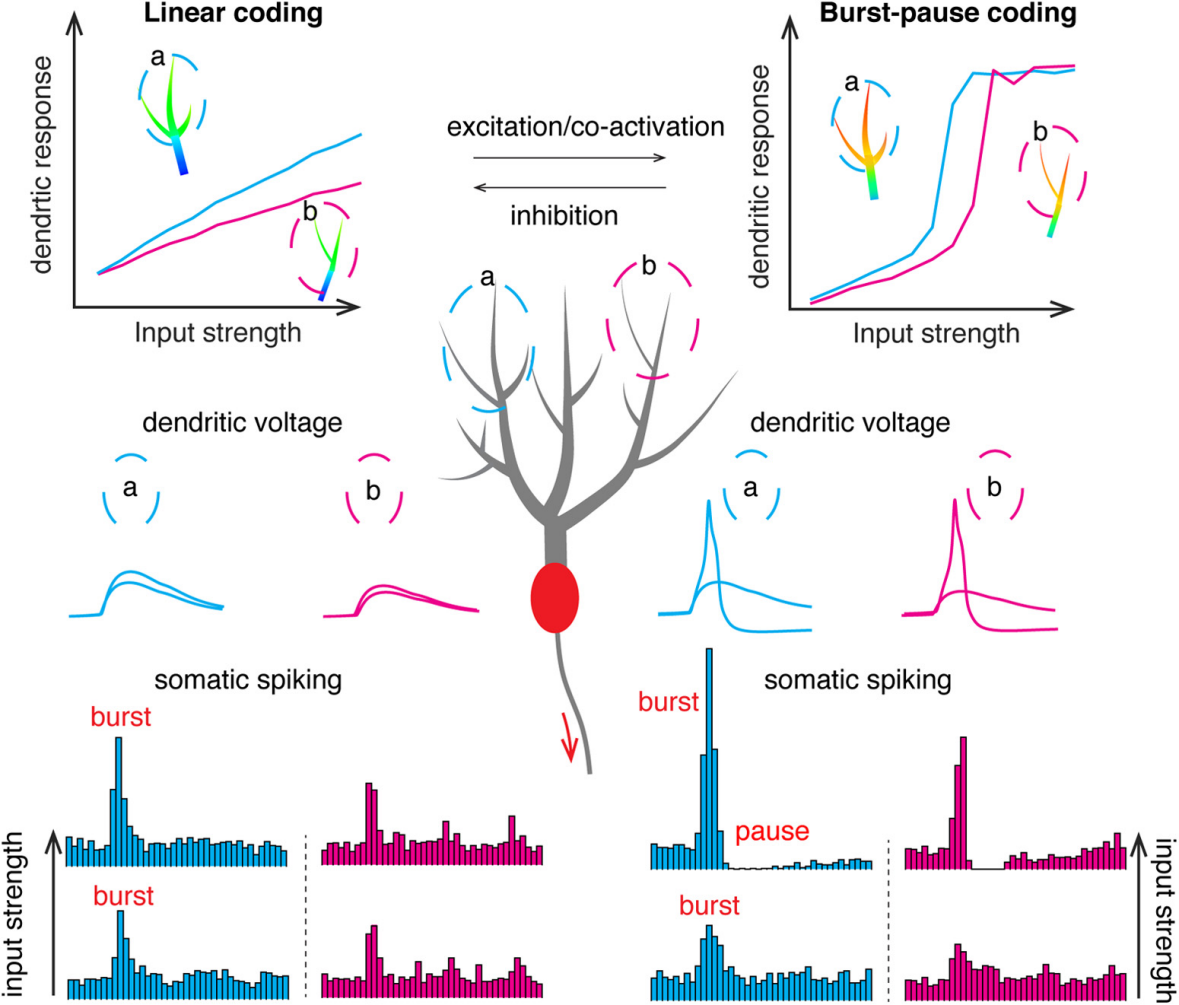
Summary of branch-dependent bimodal computations in PCs depending on factors such as voltage state, channel modulation, co-activation, and inhibition facilitating dendritic excitability, individual branches show either linear dendritic integrations or linear-step-plateau responses. Both dendritic responses decay significantly when propagating to the soma. Two branches were circled in the PC dendritic tree to show individual branch being the unit for both computations, cyan and magenta. In the range of linear coding, dendritic EPSPs only cause somatic bursts (indicated by clustered somatic spikes in PSTHs) and increase the somatic maximum spike rate with input strength; when dendritic spikes occur, they also cause reliable pauses following the bursts.

Our main findings have been summarized in Figure 11. Individual dendritic branches can either linearly integrate PF inputs or generate localized all-or-none dendritic spikes depending on the modifiable branch excitability. Both computations work in the unit of individual branches to regulate somatic output, causing somatic bursts for linear computation and somatic burst-pause sequences when dendritic spikes occur.

### Transition between linear and burst-pause computations

Cerebellar PCs have been demonstrated to linearly encode synaptic input at the ensemble level (Herzfeld et al., 2015; Chen et al., 2016) and at the single cell level (Walter and Khodakhah, 2006; Hong et al., 2016). This is surprising, considering they possess a plethora of nonlinearly voltage-gated ion channels (Migliore and Shepherd, 2002; Ohtsuki et al., 2012; Zang et al., 2018). Our model replicates the linear computation in PCs. When PF inputs are weak, high threshold P-type Ca^2+^ channels (Benton and Raman, 2009) are not activated and consequently dendritic responses are passive and linear relative to input strength (Figs. 1, 2).

Upon strong synaptic inputs, dendritic ion channels support regenerative dendritic spikes to convey information. Pyramidal neuron dendritic Na^+^ spikes can enhance orientation selectivity in the visual cortex (Smith et al., 2013). Dendritic Ca^2+^ spikes in L5 pyramidal neurons may be relevant to behavior and cognitive function (Suzuki and Larkum, 2017). Nonetheless, in cerebellar PCs, whether PF dendritic spikes occur *in vivo* and play a role in cerebellar function remain controversial. In slice experiments, PF dendritic spikes were evoked by focally stimulating a beam of PFs (Rancz and Häusser, 2006, 2010), causing strongly clustered inputs. Can focal activation of PFs also occur *in vivo* (Bower, 2010) and be powerful enough to trigger dendritic spikes? By imaging multiple neighboring cerebellar PFs receiving sensory stimuli in mice, clustered patterns of axonal activity were observed (Wilms and Häusser, 2015). Furthermore, voltage imaging in awake mice demonstrated the occurrence of localized dendritic spikes independent from CF activation (Roome and Kuhn, 2018). Theoretically, multiplexed coding underlying cerebellar behaviors, i.e., linear coding and burst-pause coding, can be implemented by two groups of PCs. However, our results show that increasing input strength shifts PCs from linear coders to burst-pause coders by evoking localized PF dendritic spikes. As a new form of coding strategy, the importance of pauses in cerebellar coding has been supported both *in vitro* (Steuber et al., 2007; Grasselli et al., 2016) and *in vivo* (Hong et al., 2016). For saccadic eye movements in monkeys, two forms of spiking patterns have been found critical for behavioral properties. Spike rate linearly encodes the eye movement velocity and pausing of somatic spikes predicts the onset of eye movements. Note that the pause signal that predicts the saccadic eye movement onset cannot be explained by CF inputs, because CFs fire only at ∼1 Hz (Hong et al., 2016). Therefore, PF dendritic spike-caused pauses described here are more likely the coding signal for timing of movement onsets. A recent theoretical study also highlights the importance of burst-pause dynamics in regulating sensorimotor adaptation (Luque et al., 2019).

PF-evoked dendritic spikes in this study are substantially different from those observed in previous work using a different PC model (De Schutter and Bower, 1994). Based on recent data (Benton and Raman, 2009; Zang et al., 2018), the activation threshold of P-type Ca^2+^ channels is high in the new model, while it was shallower in the previous model causing graded spike amplitudes.

The functional feasibility of dendritic spikes *in vivo* is constrained by their thresholds. When our model fires spontaneously, only 35 of the total ∼150,000 PF synapses converging onto a single PC are required to trigger dendritic spikes in branch 8. Even with the strongest inhibition, dendritic spikes can be triggered by just 60 PF synapses (Fig. 7). This number can be further reduced by PF bursting during sensory stimuli (Chadderton et al., 2004). Moreover, after sensory stimuli, somatic EPSPs can reach up to ∼10–17.5 mV when the soma is held at approximately −80 mV *in vivo* (estimated from Wilms and Häusser, 2015; their Fig. S1), which corresponds to at least 120 PF synapses activated in our model (Fig. 4*C*). The predicted spiking thresholds should be close to real numbers given the well-validated spiking properties of our model (Fig. 1 in this work; Zang et al., 2018, 2020) and consequently PF dendritic spikes should not be a rare signal *in vivo*. According to our simulation results (Figs. 2, 7), at the ensemble level, dendritic spikes are predicted to preferentially occur in PCs with increased firing rates during behaviors considering the balance of excitatory and inhibitory inputs (Herzfeld et al., 2015; Chen et al., 2016; Jelitai et al., 2016; Gaffield et al., 2018; Rowan et al., 2018).

### Branch-specific computation

Computation compartmentalized to specific dendrites has been demonstrated in many neurons (Branco and Häusser, 2010). In mouse motor cortex, different motor learning tasks induce Ca^2+^ spikes on different apical tuft branches in individual L5 pyramidal neurons (Cichon and Gan, 2015). Combined with precise inhibition, postsynaptic Ca^2+^ signals can even be controlled within individual spines in L2/3 pyramidal neurons (Chiu et al., 2013). Previously we uncovered that PCs show branch-specific responses to CF input because of their typical morphology (Zang et al., 2018). The heterogenous excitability can be further tuned by the modulation of dendritic ion channels such as SK2 and Kv4 currents (Ohtsuki et al., 2012; Zang et al., 2018).

PF-triggered dendritic spikes are all-or-none and spatially constrained (Fig. 1). The different electronic distances of individual branches to the soma make individual branches as computational units (Figs. 4, 8), which challenges the traditional view of PCs as point neurons. The question of how many dendritic compartments are needed to capture a neuron's information processing capacity (Häusser and Mel, 2003) is critical for biologically based artificial intelligence (Guerguiev et al., 2017). Although we manually defined 22 branches in the model, they may not represent the actual functional units that individual PCs use to process information. First, the gain curves of some branches overlap with each other. Second, depending on the clustering degree of PFs, branches and corresponding burst-pause units may be further divided. Most of all, we have shown that a dendritic branch can transition between linear coder and burst-pause coder by hyperpolarization (Fig. 2), between-branch interaction (Fig. 5), ionic channel modulation (Fig. 6), and synaptic inhibition (Fig. 7), all of which make the number of functional units harder to estimate. It is more likely that the number of computational units for either linear coding or burst-pause coding is not fixed. Depending on functional needs, the above-mentioned modulation factors shift dendritic spike thresholds to favor either linear computation or burst-pause computation to increase the efficiency of dendritic information processing (Fig. 11).

### Degenerate function of PF dendritic spikes

Degeneracy and complexity are prominent properties of many biological systems (Edelman and Gally, 2001; Zang and De Schutter, 2019). Apart from the above-mentioned burst-pause coding behavioral properties, PF dendritic spikes trigger substantial Ca^2+^ influx in the confined branch, which depresses PF synapses in long-term (Hartell, 1996) and short term (Kreitzer and Regehr, 2001; Rancz and Häusser, 2006). Therefore, PF dendritic spikes can play a degenerate role in cerebellum-associated function. Depending on the context, on the one hand, they can encode behavioral properties by generating a burst-pause sequence to regulate the spiking of downstream neurons; on the other hand, they can supplement CFs as instruction signals to initiate cerebellar learning (Gallimore et al., 2018). Actually, PF dendritic spikes may be a better candidate signal for context-dependent learning, since PF input encodes the ongoing behavioral states (Chen et al., 2017).

Taken together, individual PCs can shift between linear coders and burst-pause coders to implement branch-dependent multiplexed coding to increase cerebellar coding and learning capacity.
